# Application of Silicon Influencing Grain Yield and Some Grain Quality Features in Thai Fragrant Rice

**DOI:** 10.3390/plants13101336

**Published:** 2024-05-12

**Authors:** Phukjira Chan-in, Sansanee Jamjod, Chanakan Prom-u-thai, Benjavan Rerkasem, Joanne Russell, Tonapha Pusadee

**Affiliations:** 1 Plant Genetic Resource and Nutrition Lab (CMUPNLab), Division of Agronomy, Department of Plant and Soil Sciences, Faculty of Agriculture, Chiang Mai University, Chiang Mai 50200, Thailand; phukjira_c@cmu.ac.th (P.C.-i.); sansanee.j@cmu.ac.th (S.J.); chanakan.p@cmu.ac.th (C.P.-u.-t.); 2 Lanna Rice Research Center, Chiang Mai University, Chiang Mai 50200, Thailand; benrerkasem@gmail.com; 3 Cell and Molecular Sciences, The James Hutton Institute, Dundee DD25DA, UK; joanne.russell@hutton.ac.uk; 4 Agrobiodiversity in Highland and Sustainable Utilization Research Group, Faculty of Agriculture, Chiang Mai University, Chiang Mai 50200, Thailand

**Keywords:** fragrant rice landraces, Si fertilizer application, 2AP biosynthesis pathway, fragrant genes, gene expression

## Abstract

Silicon (Si) is a beneficial nutrient that has been shown to increase rice productivity and grain quality. Fragrant rice occupies the high end of the rice market with prices at twice to more than three times those of non-fragrant rice. Thus, this study evaluated the effects of increasing Si on the yield and quality of fragrant rice. Also measured were the content of proline and the expression of the genes associated with 2AP synthesis and Si transport. The fragrant rice varieties were found to differ markedly in the effect of Si on their quality, as measured by the grain 2AP concentration, while there were only slight differences in their yield response to Si. The varieties with low 2AP when the Si supply is limited are represented by either PTT1 or BNM4 with only slight increases in 2AP when Si was increased. Si affects the gene expression levels of the genes associated with 2AP synthesis, and the accumulation of 2AP in fragrant rice mainly occurred through the upregulation of *Badh2*, *DAO*, *OAT*, *ProDH*, and *P5CS* genes. The findings suggest that Si is a potential micronutrient that can be utilized for improving 2AP and grain yield in further aromatic rice breeding programs.

## 1. Introduction

Silicon (Si) is a non-essential element that has been discovered to be beneficial for the growth and development of horticultural and agricultural crops [1,2]. In *Gramineae* crops, especially wheat, rice, and sugarcane, Si has been reported to enhance the growth and biomass, yield, and grain quality [3,4]. Several studies have reported that Si fertilizer has direct or indirect effects on plant growth and development. Si fertilizer enhances cell wall strength, reduces lodging, and enhances resistance to abiotic and biotic [5,6,7,8]. Rice is indeed a known accumulator of silicon by taking up and accumulating large amounts of silicon from the soil through the roots and depositing it in the plant tissues [9,10]. Plant roots uptake Si via active, passive, and rejective transport that differ among plant species. In three modes of Si uptake, it has been suggested that the active mechanisms were the best Si transport across the cell membrane [8,9,10,11]. The lateral roots uptake Si from the soil by active and passive mechanisms, then transport it passively to shoot through the xylem via a transpiration stream, where it accumulates in the cell wall of leaves, stems, and other plant tissues [5,11,12,13,14,15] Several studies have identified genes involved in regulating Si transporters in rice that uptake and distribute Si from the root to other plant parts (i.e., the cell wall of leaves, stems, and hulls). The Si accumulation in the shoot ranges from 1.5 to 10% Si concentrations in the plant’s dry weight, depending on the species [5,12,13]. There are several genes known as *Lsi* genes involved in different transport mechanisms throughout the plant. The genes involved in the root transport are *OsLsi1* and *OsLsi2*, for Si influx and efflux, respectively [14,16,17,18,19]. Additionally, the study also found that *OsLsi2* and *OsLsi3* are involved in the active efflux transport of silicon from the shoot into panicles [20,21,22,23,24]. *OsLsi6* is highly expressed in node-1 below the panicles in the reproductive growth stage of rice as an influx transporter and a homolog of *OsLsi1*, responsible for unloading the Si into the xylem vessels via transport in the transpiration stream for distributing Si to the panicle and grain [24,25,26,27]. In addition, the study of Wangkaew et al. [28] found that *OsLsi6* was not detected in the roots at all stages, but the highest expression was observed in the node at the booting and flowering stages. Furthermore, Chaiwong et al. [29] also reported that *OsLsi6* can be utilized to enhance rice production by promoting the development of caryopsis. 

Rice (*Oryza sativa*) is an important crop globally as the staple food for more than half of the world’s population, particularly in Asia [30,31]. Fragrance rice or aromatic rice refers to a small but special group of rice varieties that present an aroma on the grain with a “popcorn-like” or “pandan-like” flavor [32,33]. Fragrance in rice is an essential factor that contributes to its desirability and value in both domestic and international markets. The rice with the highest prices in the world market are Indian and Pakistan Basmati, followed by Thai Hom Mali and Cambodian Phka Malis [34]. The premium prices, at 2–3 times that of ordinary non-aromatic rice, are due to their aroma and unique grain quality characteristics preferred by high-income consumers [35,36,37]. The key compound in fragrant rice’s aroma is 2-acetyl-1-pyrroline (2AP), which is produced under the control of the recessive aroma gene [38,39,40,41]. L-proline, L-ornithine, hydroxyproline, and glutamic acid have been identified as possible precursors for the formation of 2AP in aromatic rice via a polyamine pathway with Δ1-Pyrroline-5-carboxylate synthetase (P5CS; coded by *P5CS* gene), proline dehydrogenase (ProDH; coded by *PRODH* gene), and ornithine aminotransferase (OAT coded by *OAT* gene) associated with the rise of Δ1-pyrroline. Moreover, ornithine diamine oxidase (DAO coded by *DAO* gene) can convert ornithine to polyamine putrescine, which is then transformed into 1-pyrroline [42,43,44,45] and, subsequently, the loss function of the betaine-aldehyde dehydrogenase (BADH2 coded by *BADH2* gene) leads to aroma formation in fragrant rice [46,47,48,49,50]. However, another proposed pathway for 2AP biosynthetic is the glycolysis pathway with methylglyoxal as a precursor [51]. 

Furthermore, Si has been associated with improving rice grain quality by increased milling quality, reduced chalkiness, and enhanced cooking and eating characteristics [52,53,54]. Applying Si fertilizer increased the 2AP content, Si and proline contents in the leaf and grain in fragrant rice, the growth, and yield due to increased photosynthetic activities [6]. Likewise, the 2% *w/v* of foliar Si application improved grain yield, yield traits, and 2AP content in Thai jasmine rice by enhancing panicle weight, the number of fertile seeds, grain fertility percentage, the number of panicles, total grain weight, and harvest index [55]. Moreover, several studies have demonstrated the significant effects of combining fertilizers such as nitrogen-silicon (N-Si) fertilization on grain yield and lodging resistance in fragrant rice. This effect is attributed to the increased activities of N metabolism enzymes and the accumulation of N and Si in various parts of the rice plant. Notably, the high accumulation of N and Si directly regulates the 2AP content or indirectly influences the antioxidant response parameters, ultimately promoting the formation of 2-AP [56]. Similarly, the application of Se-Si treatment in fragrant rice led to the regulation of plant growth. Moreover, this treatment improved the grain 2AP content by regulating the metabolism of proline, pyrroline-5-carboxylate (P5C), and γ-aminobutyric acid (GABA) [57,58].

In Thailand, where rice has a social as well as economic importance, fragrant rice accounts for approximately one-third of the annual production, with some 70% grown from Khao Dawk Mali 105 (KDML105), a photoperiod-sensitive variety with a traditional plant type [59]. There are numerous other fragrant rice varieties, including several landraces, in Thailand’s genetically highly diverse local rice germplasm, some of which are differentiated from the mega variety KDML105 in their grain morphology, cooking qualities, aroma, and allelic variation of the fragrant gene *Badh2* [60]. One of these, BNM4 (Buer Ner Moo 4), was evaluated in its response to Si, in comparison with KDML105, and a modern fragrant variety, Pathum Thani 1 (PTT1). However, Thai landraces have not yet been exploited by comparing them to the successful KDML105 and a modern variety, PTT1. Therefore, the objectives are to examine the yield and quality response to different silicon applications and to determine which genes are expressed during the important growing stages and aroma production. Thus, the results are expected to increase our understanding of how rice yield and the quality of fragrance are affected by Si nutrition, forming a basis for the development of Si fertilizer management for fragrant rice.

## 2. Results

### 2.1. Grain Yield and Yield Component

**Figure 1:**
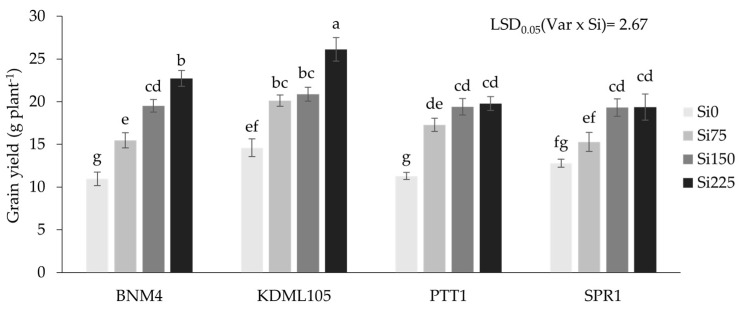
Grain yield of four rice varieties grown at different silicon fertilizer applications. Bars represent standard error of mean. Different lowercase letters above bars indicate least significant differences at *p <* 0.05. (n = 24).

The grain yield of the four rice varieties was increased by Si, but with different responses to the Si rates (Figure 1). All four rice varieties had the lowest grain yield at Si0. The yield of BNM4 and KDML105 increased with increasing Si to Si225, while the yield of PTT1 and SPR1 reached the maximum at Si150.

The rice varieties were affected differently by Si in their plant height and percentage of filled grain (*p* < 0.001), while there was a general trend of small increases in thousand-grain weight with increasing Si. Additionally, Si had no significant effect on the number of tillers and panicles nor the effect of variety and the interaction between variety and Si (Table 1). The highest plant height was found in KDML105 in all silicon applications, which increased with increasing Si to Si225. In BNM4 and SPR1, plant height increased with increasing Si to Si75, but at Si150, the plant height decreased. Meanwhile, the plant height of PTT1 decreased with increasing Si to Si225. Applying silicon fertilizer continued to increase the percentage of filled grain in KDML105 and SPR1. In BNM4, the percentage of filled grain at Si75 decreased below that with Si0 and increased with increasing Si75 to Si225. The thousand-grain weight increased with increasing Si to Si225 in all varieties. Among the varieties, the highest thousand-grain weight was found in BNM4, whereas KDML105 and SPR1 had the lowest thousand-grain weight.

### 2.2. Silicon Concentration

**Figure 2:**
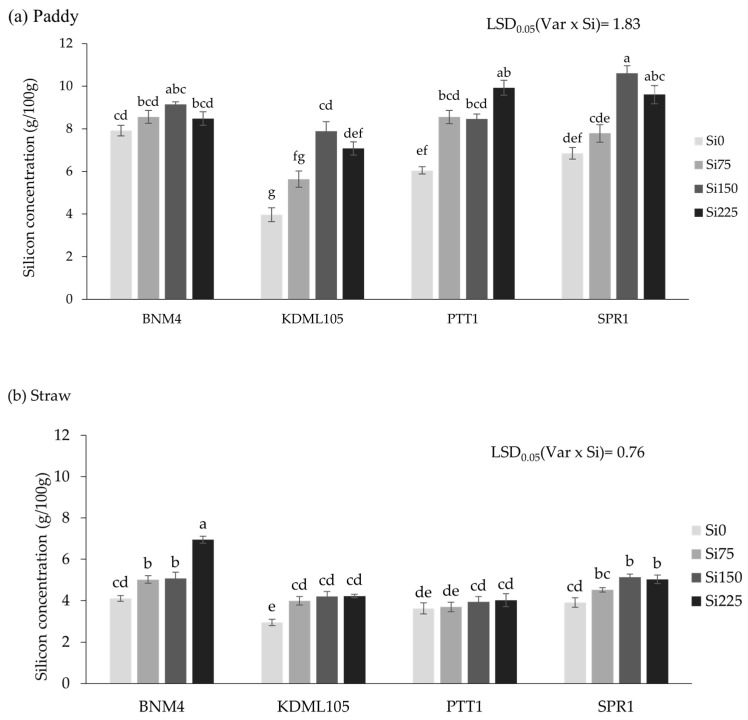
Silicon concentration in the paddy (**a**) and straw (**b**) at maturity of four rice varieties grown with different silicon fertilizer applications. Bars represent standard error of mean. Different lowercase letters above bars indicate least significant differences at *p <* 0.05 (n = 6).

The silicon application increased the silicon concentration in the paddy and straw in the four rice varieties differently (*p* < 0.001) (Figure 2). When no Si was applied, the rice varieties accumulated Si in the paddy at different concentrations, with the highest in BNM4, followed by SPR1 and PTT1, and the lowest in KDML105 with only half the concentration in BNM4. The paddy Si was increased only slightly by increasing the Si rate in BNM4 and more strongly in the other three varieties. Maximum paddy Si was reached at Si150 in KDML105 and SPR1, and at Si225 in PTT1 (Figure 2a). The straw Si levels were generally much lower than the paddy Si and responded to increasing Si among the rice varieties differently from the paddy Si (Figure 2b). At Si0, for example, the straw Si in BNM4 was only half of its paddy Si. Increasing the Si rate increased the straw Si strongly in BNM4 but had little or no effect in the other three varieties.

### 2.3. Proline Concentration

**Figure 3:**
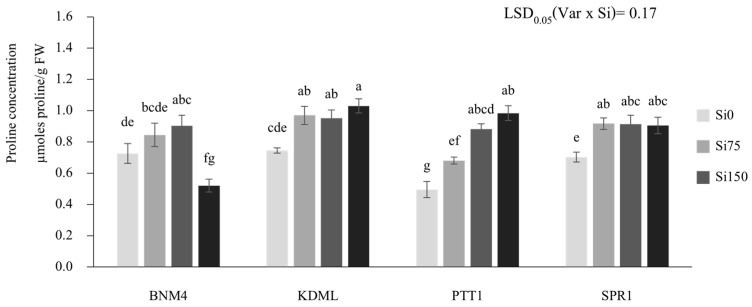
Proline concentrations in the leaves in response to different silicon fertilizer applications at flowering stage of four rice varieties. Bars represent standard error of mean. Different lowercase letters above bars indicate least significant differences at *p <* 0.05 (n = 3).

Silicon fertilizer applications significantly affected proline concentrations differently between rice varieties (*p* < 0.05) (Figure 3). In BNM4, the proline concentration increased with increasing Si to Si150, but at Si225, the proline decreased below that with Si0. The proline in PTT1, on the other hand, continued to increase with Si to Si225. KDML105 and non-fragrant SPR1 responded to Si in the same way, in that there was a significant increase in the proline concentration from Si0 to Si75, with little or no additional effect at higher rates.

### 2.4. Grain Quality

#### 2.4.1. Aroma–2AP Concentration

**Figure 4:**
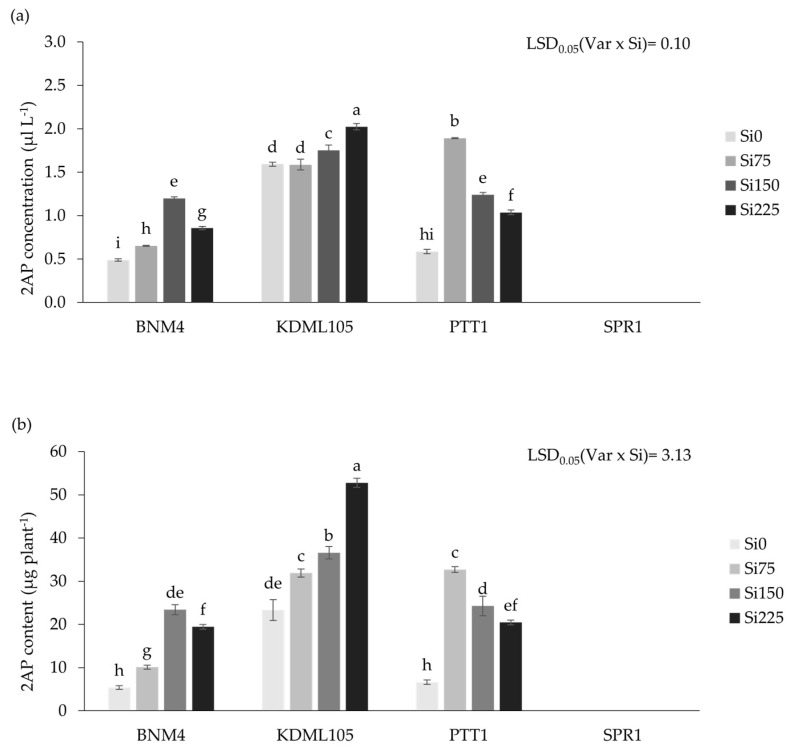
The 2-acetyl-1-Pyrroline (2AP) concentration (**a**) and content (**b**) in grain of three fragrant rice varieties (SPR1 is non-aromatic rice) grown at different silicon fertilizer applications. Bars represent standard error of mean. Different lowercase letters above bars indicate least significant differences at *p <* 0.05 (n = 3).

The three fragrant rice varieties responded differently to increasing rates of Si application in the 2-acetyl-1-pyrroline (2AP) concentration of their milled grain (white rice), while no 2AP was detected in the non-fragrant SPR1 (Figure 4a). At Si0, the 2AP concentration of KDML105 was triple that in PTT1 and BNM4. Increasing Si increased the 2AP concentration in all three varieties but with varying responses. In KDML105, the 2AP concentration increased significantly with higher rates up to the maximum at Si225. In PTT1, the 2AP concentration increased most strongly to the level approaching the maximum in KDML105, but with significant declines at higher Si rates. The application of Si also increased the 2AP concentration in BNM4, but much less strongly than KDML105 and PTT1, reaching the maximum at Si150 at a low 2AP concentration only two-thirds of that in KDML105 at Si0 (Figure 4a).

The content of 2-acetyl-1-pyrroline (2AP), calculated from the harvested yield as the 2AP yield, was affected by the Si fertilizer rate differently among the rice varieties (*p* < 0.05) (Figure 4b). Compared to the control treatment, applying silicon fertilizer at 75 and 150 ppm increased the 2AP content in BNM4 by 87.8% and 131.5%, respectively, but decreased at 225 ppm silicon fertilizer by 16.9%. In KDML105, applied silicon fertilizer at 75, 150, and 225 ppm increased 2AP content by 36.6%, 14.8%, and 44.3%, respectively, compared with the Si0 treatment. On the other hand, applying silicon fertilizer in PTT1 increased 2AP content when compared with the control treatment at 75 ppm content by 392.9% while 2AP decreased at Si150 and Si225 by 25.8% and 15.7%, respectively. The highest 2AP content was obtained at 225 ppm in KDML105 (52.8 µg/plant), while SPR1 was non-fragrant rice and the 2AP content was zero (not detected). 

#### 2.4.2. Amylose Content (%)

**Figure 5:**
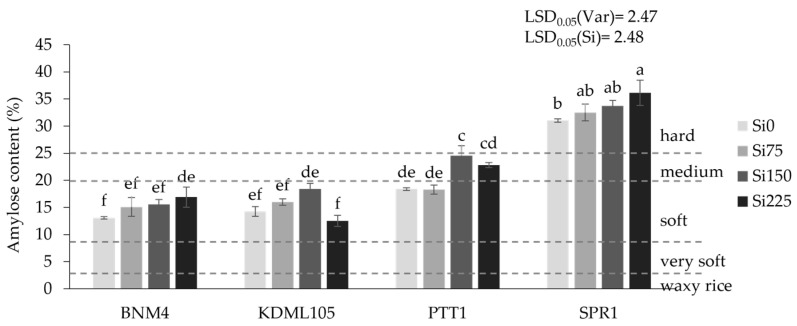
Amylose content (%) of four rice varieties grown at different silicon fertilizer applications. Amylose content between 0 and 2% is defined as waxy rice, between 3 and 9% as very soft rice, between 10 and 19% as soft rice, between 19 and 25% as medium rice, and more than 25% as hard rice [61,62]. Bars represent standard error of mean. Different lowercase letters above bars indicate least significant differences at *p <* 0.05 (n = 3).

There was no interaction between variety and Si in their effects on the rice grain amylose percentage, although there were significant differences among the varieties and the Si effects (*p* < 0.05) (Figure 5). At 15%, KDML105 and BNM4 were lowest in amylose, followed by PTT1 at 20%, and highest in SPR1 at 33%. Although significant, the general effects of Si on amylose were slight.

### 2.5. Gene Expression

**Figure 6:**
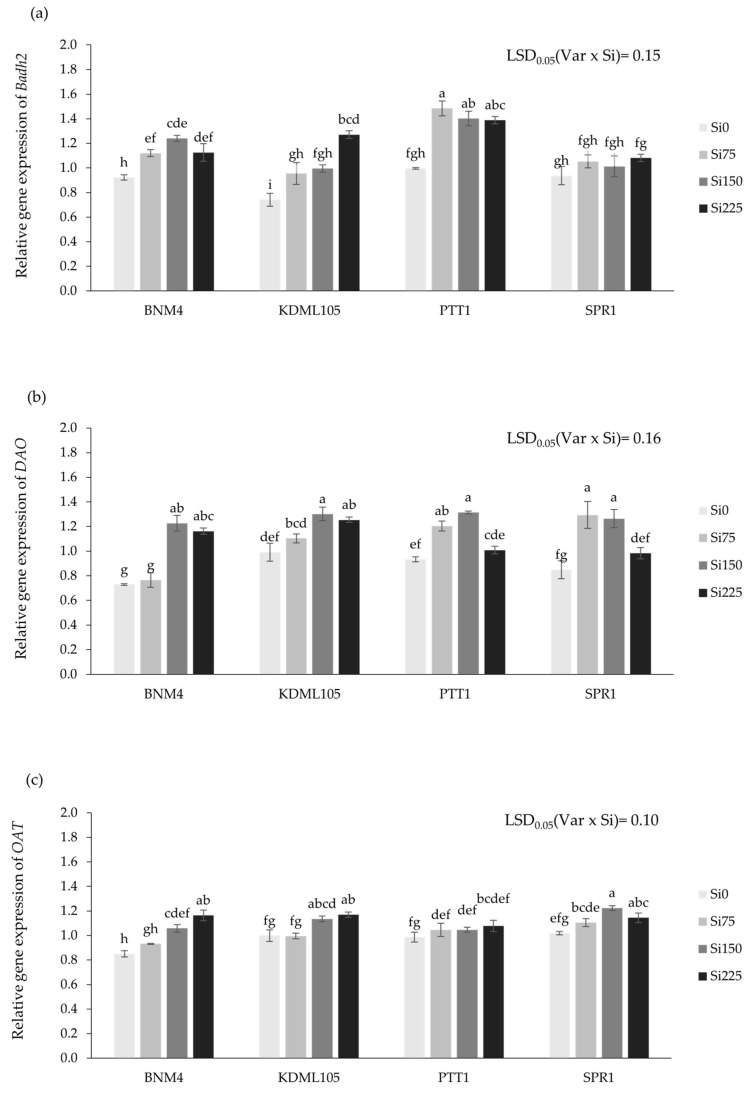

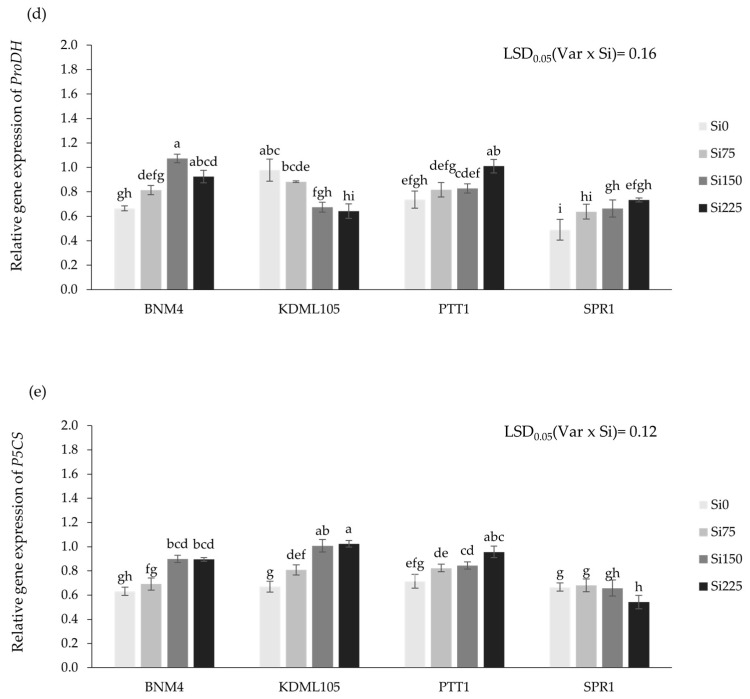
Relative gene expression of *Badh2* (**a**), *DAO* (**b**), *OAT* (**c**), *ProDH* (**d**), and *P5CS* (**e**) genea of four rice varieties grown at different silicon fertilizer applications using real-time quantitative RT-PCR (qRT-PCR) analysis. Bars represent standard error of mean. Different lowercase letters above bars indicate least significant differences at *p <* 0.05 (n = 3).

The expression levels of *Badh2*, *DAO*, *OAT*, *ProDH*, and *P5CS* in 5DAF in panicle and *OsLsi6* in the first node of rice plants were significantly affected by Si fertilizer application between rice varieties (Figure 6). 

The expression levels of the *Badh2* gene in BNM4 and PTT1 significantly increased by 21.4–34.5% and 39.3–48.8%, respectively, compared to Si0. KDML105 increased progressively with increasing Si to 225 ppm by 28.9–71.5%, but no significant difference in the level of gene expression at Si75 and Si150 was observed. Meanwhile, SPR1 was unresponsive to Si. The highest expression level of *Badh2* was found in PTT1 under Si75, Si150, and Si225, respectively (Figure 6a). In BNM4 under Si75, there was no significant difference in the expression level of the *DAO* gene with Si0, while under the application of Si150 and Si225, they increased by 31.2 and 26.4% from Si0. The expression of *DAO* in the KDML105 gene increased progressively with increasing Si by 11.2–31.2%. PTT1 and SPR1 displayed significant expression levels of *DAO* gene increases when applied to Si75 and Si150 (29.0, 40.8, 52.4, and 49.0%, respectively) (Figure 6b). The expression of the *OAT* gene increased progressively with increasing Si to 225 ppm by 9.6–36.9% in BNM4. In KDML105 and SPR1, there were significant levels of *OAT* gene expression increases with 75 to 225 ppm. SPR1 had no significant difference effect of Si on *OAT* gene expression (Figure 6c). *ProDH* expression was progressively enhanced in all rice varieties except KDML105, which decreased gene expression from Si0 by 10.7, 45.1, and 52.3% when applied to Si75, Si150, and Si225, respectively (Figure 6d). Upon increasing the application of Si, the expression level of *P5CS* increased in BNM4, KDML105, and PTT1, but not SPR1, which was not significant in *P5CS* gene expression (Figure 6e). 

**Figure 7:**
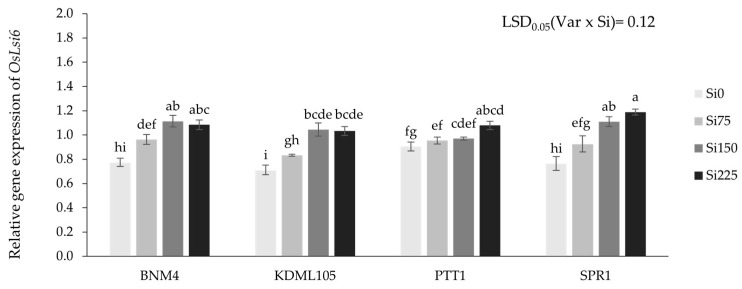
Relative gene expression of *OsLsi6* gene of four rice varieties grown with different silicon fertilizer applications using real-time quantitative RT-PCR (qRT-PCR) analysis. Bars represent standard error of mean. Different lowercase letters above bars indicate least significant differences at *p <* 0.05 (n = 3).

Applying Si fertilizer increased the expression level of *OsLsi6* in all rice varieties. The highest expression level of *OsLsi6* was observed in BNM4 and SPR1 under Si150 and Si225, respectively. PTT1 showed a significant increase in the expression only with Si 225 ppm, while KDML105 showed no significant difference in gene expression under Si0 and Si75 but applying Si from 75 to 150 enhanced the level of *OsLsi6* expression by 25.3% (Figure 7).

The correlation analysis between proline, 2AP concentration, and the relative gene expression levels (RQ) of *Badh2*, *DAO*, *OAT*, *ProDH*, and *P5CS* genes in 5 DAF of four rice varieties is shown in Table 2. The 2AP concentration was positively correlated with the RQ*Badh2* gene (r = 0.30; *p* < 0.05), RQ*DAO* gene (r = 0.34; *p* < 0.05), RQ*P5CS* gene (r = 0.68; *p* < 0.001), and RQ*ProDH* gene (r = 0.38; *p* < 0.01). There was also a significant positive correlation between the proline concentration with the RQ*OAT* gene (r = 0.41; *p* < 0.05), RQ*P5CS* gene (r = 0.44; *p* < 0.05), and RQ*ProDH* (r = 0.34; *p* < 0.05), between the RQ*Badh2* gene and the RQ*DAO* gene (r = 0.34; *p* < 0.05) and RQ*P5CS* gene (r = 0.44; *p* < 0.01), and between the RQ*DAO* gene and the RQ*OAT* gene (r = 0.59; *p* < 0.001) and RQ*P5CS* gene (r = 0.45; *p* < 0.001).

## 3. Discussion

### 3.1. Yield Response and Expression of Si Transport Gene

Si fertilizer applications were found to have variable effects on proline concentrations in leaves at the flowering stage. Proline concentration increased after the treatments, except in BNM4 at Si225, which decreased. In all varieties, the highest grain yield was obtained with increasing levels of silicon fertilizer, with KDML105 outyielding the other lines across all applications. The increased grain yield can be attributed to an improved increase in the percentage of filled grain while the number of tillers and panicles per plant did not show any significant difference. However, previous studies found that silicon application increased rice yield, especially the number of tillers and panicles per plant and the percentage of filled grains [63,64]. The study by Detmann et al. [65], which compared a wild type and mutant, showed significant differences between the genotypes. The Si fertilizer not only improved crop yield but also affected photosynthesis, with increased mesophyll conductance and photosynthetic activity. 

The effect of Si fertilizer on Si concentrations in different plant parts, with differences between the paddy and straw among different rice varieties and Si levels, was observed. However, due to the growing conditions in the soil, the roots were not examined in this study. This variability highlights the intricate mechanisms governing Si uptake, transport, and distribution within the plant. Previous studies demonstrated that Si accumulated much more in the rice husk during the grain development stages [66]. The variability in response could be explained by differences in Si uptake, transport, and Si accumulation among rice varieties, which all depend on its ability to take up the elements from the soil via the root [27] and are related to the expression levels of Si transporter genes [14,15]. The results from the gene expression analysis of *OsLsi6* in the nodes of four rice varieties increased differently with rising Si fertilizer rates and related to a positive correlation with Si concentration in paddy and straw. Likewise, the recent study of Chaiwong et al. [64] reported that the highest expression *OsLsi6* was obtained at the highest Si rate and demonstrated that the Si concentration in rice plants and Si total uptake were related to the expression of the *OsLsi6* gene. Additionally, the study of Lavinsky et al. [67] revealed an increase in the expression level of *OsLsi6* when Si fertilizer was applied at the reproductive stage, potentially contributing to the higher Si levels in the rice panicles. It has also been reported that *OsLsi6* functions as a Si transporter that is responsible for unloading and the distribution of Si from the xylem to accumulate in the leaf and grains [11,14,21,68]. Interestingly, a study by Wangkaew et al. [28] revealed that SPR1 accumulated more Si in its transpiring organs, including the leaves and husks, and exhibited higher expression of *OsLsi1*, *OsLsi2*, and *OsLsi6* compared to PTT1. This indicates that while PTT1 has the ability to absorb silicon, it may respond to silicon or utilize it, which could potentially lead to reduced aroma or other effects on various aspects or mechanisms within the plant.

### 3.2. Effects of Silicon on the Aroma (2AP)

Without Si application, the higher 2AP concentration in the grain of KDML105 compared to PTT1 and BMN4 is in agreement with previously reported findings [69]. Aroma is the primary determinant of Thai Hom Mali rice, while other grain quality features such as head rice yield, grain translucency, and vitreousness are satisfied [70]. The high 2AP concentration found here in KDML105 in Si0 suggests that it is likely to perform well with respect to aroma in soils low in available Si. This, along with KDML105’s ability to maintain high head rice yield when nitrogen is limited [71], is likely to have contributed to the variety’s popularity in Thailand, accounting for 70% of the country’s fragrant rice production, with a total area of 4.5 million ha annually (computed from data in OAE [72]). The lower 2AP concentration of the rice varieties PTT1 than KDML105 has been consistently shown under a wide range of conditions [69,73]. The almost 4-fold increase in the 2AP concentration in PTT1 with a low Si rate (Si75) found in the present study suggests that the application of Si fertilizer may be a means of quality improvement in this high-yielding, photoperiod-insensitive fragrant rice variety. Although the 2AP concentration in BMN4 approached that of KDML105 when grown in the highlands where it originated [69], Si fertilizer was less effective in increasing the 2AP concentration in the landrace line of fragrant rice. 

The correlation observed in this study between proline, the 2AP concentration, and gene expression associated with 2AP biosynthesis indicates that the expression levels of *Badh2*, *DAO*, *ProDH*, and *P5CS* genes were linked to 2AP production and the expression of *OAT*, *P5CS*, and *ProDH* in relation to the proline concentration in the polyamine pathway. In general, *OAT*, *P5CS*, and *ProDH* are the genes that code the enzyme catalysis in the aroma pathway, a vital step in the formation of Δ1-pyrroline-5-carboxylate (P5C) using proline, glutamic acid, and ornithine as precursors with P5C converted into △1-pyrroline [42,74,75]. Furthermore, the non-functionality of the *Badh2* gene leads to the synthesis of 2AP in scented rice, resulting in the accumulation of γ-amino butyraldehyde (GABald) and ultimately to the formation of 2AP [76,77]. Another process involving synthesizing 2AP was regulated by *BADH2* and *DAO* genes. *DAO* transforms putrescine into GABald, which further converts to Δ1-pyrroline or synthesizes GABA, depending on the absence or presence of the functional BADH2 enzyme [78,79]. Nevertheless, this study demonstrates that the increase in the 2AP concentration was not associated with the proline concentration. Thus, there is likely another precursor involved in the biosynthesis of 2AP. The study of Huang et al. [42] also suggested that methylglyoxal in the glycolysis pathway might be a precursor for 2AP biosynthesis and found a strong positive correlation between methylglyoxal and 2AP accumulation. While all genes may not be directly associated with fragrance traits, they can still be utilized in studies examining the responses of rice to fertilizers. However, further investigation is necessary at the transcript level to provide a comprehensive understanding. Therefore, in future studies, the intermediates in 2AP biosynthesis pathways should be more extensively explored.

## 4. Materials and Methods

### 4.1. Plant Culture and Experimental Design

A factorial experiment with 4 rice varieties and 4 rates of Si was conducted during the wet season of 2022 (from June to November) at Chiang Mai University, in Northern Thailand. Four 14-day-old seedlings of 3 fragrant rice varieties, a landraceBNM4 [52], KDML105 (traditional plant type), PTT1 (high yielding, modern plant type), and one non-fragrant, high-yielding variety, Suphan Buri 1 (SPR1), were each grown in a plastic pot (30 cm-diameter, 25 cm-deep), containing 15 kg of a loamy sand soil, with 2.23% organic matter, 0.12% total nitrogen (%), 38.01 mg kg^−1^ available phosphorus (P), 173.79 mg kg^−1^ exchangeable potassium (K), pH 5.46, and electrical conductivity of 2.58 dS m^−1^, with 5 plants per pot and 2 pots per variety. Silicon was applied at the maximum tillering stage and booting stage as a sodium metasilicate pentahydrate (Na_2_SiO_3_·5H_2_O) at 0, 75, 150, and 225 mg kg^−1^ soil (designated as Si0, Si75, Si150, and Si225, respectively). The pots were arranged in a factorial randomized complete block design with 3 replications (n = 30 in each treatment). The plants were grown as wetland rice, with 15 cm of water maintained above the soil surface during the whole crop-growth period. Each pot underwent applications of 60 kg N ha^−1^ (46% N), 21 kg P_2_O ha^−1^, and 15 kg K_2_O ha^−1^, which was split twice equally at 7 days after the transplanting and maximum tillering stages. 

### 4.2. Yield and Yield Components

Samples were manually harvested at the maturity stage, and four plants per pot were harvested and evaluated for yield and yield components (plant height, number of tillers plant^−1^, number of panicles plant^−1^, panicle length (cm), and percentage of filled grain). The grain yield was carefully threshed and air-dried to measure the thousand-grain weight. Some grain samples were stored at minus 20°C for sensory tests and 2AP analysis. Other grain samples were kept at room temperature before processing for grain-quality analysis. The husk, outer covering of the rice seed, and straw samples were oven-dried at 75 °C for 72 h before Si concentration analysis.

### 4.3. Silicon Concentrations Analysis

The silicon concentration was determined in the paddy, as very little Si accumulates in the rice kernel [29], and straw. Approximately 10 g of dried samples were ground into a fine powder. The analysis of the Si concentration was conducted using the autoclave-induced digestion method [80,81]. Then, 0.1 g subsamples were analyzed and digested in 50% sodium hydroxide. The analysis for Si concentration was conducted using a spectrophotometer at 650 nm. Silicon was calculated based on the standard curve.

### 4.4. Proline Concentration

At maximum tillering and booting stages, seven days after applying silicon fertilizer and at the 50% heading (flowering) stage, a second young leaf blade on the main stem was collected and determined according to the method described by Bates et al. [82]. The absorbance was measured at 520 nm. The proline concentration was determined by comparison with a standard curve and expressed as µmoles proline g^−1^ of fresh weight.

### 4.5. Grain Quality

Aroma, as represented by the 2AP concentration, and amylose content, two key features of Jasmine-type aromatic rice, were determined.

#### 4.5.1. Aroma–2AP Concentration

Ten grams of milled grain (1 year of storage at −20 °C as white rice) of each sample were ground into a powder and sieved through a 35-mesh sieve. Then, 1.0 g of rice powder was placed into a headspace vial containing 1.0 µL of 500 ppm 2,4,6-trimethylpyridine (TMP) as an internal standard. The headspace vials were immediately sealed with PTFE/silicone septa and aluminum caps before analysis using static headspace-gas chromatography coupled with a nitrogen/phosphorus detector (SHS-GC–NPD) [73] at Salana Organic Village (Social Enterprise) Company Limited (Nakhon Pathom, Thailand).

#### 4.5.2. Amylose Content

Amylose content was determined on the rice flour using the conventional method [61,83]. The absorbance of the solution was measured at 620 nm with a spectrophotometer. The determination of amylose content was calculated according to a standard curve developed using different ratios of amylose blends.

### 4.6. Gene Expression Analysis

Plant samples were collected for gene expression analysis 5 days after flowering (DAF). Fresh tissues of the first node below the panicle and panicle were collected and ground in liquid nitrogen. RNA was extracted using the TRIzol **^®^** reagent following the protocol provided by the manufacturer (Invitrogen, Thermo Fisher Scientific, Waltham, MA, USA) and treated with DNase. Approximately 1 µg of total RNA was used for first-strand cDNA synthesis using a RevertAid first-strand cDNA synthesis Kit (Thermo Fisher Scientific, MA, USA). Gene expression levels of *Badh2*, *DAO*, *OAT*, *ProDH*, *P5CS*, and *OsLsi6* were analyzed using Quantitative real-time RT-PCR (qRT-PCR) using gene-specific primers from the previous studies as shown in Table 3. PCR amplification was performed in a 20 μL final volume containing 10 μL of the 2× SensiFASTTM SYBR green No-ROX kit (Bioline Reagents Ltd., London, UK), 0.3 μL each of 10 µmol forward and reverse primers, and 8.4 μL of 100 ng cDNA as a template. The RT-PCR condition was determined by denaturing at 95 °C for 2 min followed by 40 cycles at 95 °C for 30 s, 60 °C primer annealing for 30 s, and extension at 72 °C for 30 s. Then the temperature was raised gradually by 0.5 °C every 10 s to perform the melt-curve analysis. The PCR reactions were performed on QIAquant 96 5plex instruments and operated via the Q-Rex Software (Qiagen, Hilden, Germany). The relative quantities of each amplified product in the samples were assessed using the comparative ΔΔCt method. Duplicate measurements were averaged, and the mean values were used to adjust Ct values for further calculations.

### 4.7. Statistical Analysis

Statistical analysis of the data for different rice varieties and treatments of Si fertilization application in this study were examined using analysis of variance (ANOVA, New Providence, NJ, USA) via the Statistic8 program (analytical software, SXW, Tallahassee, FL, USA). Least significant differences (LSD) at *p* < 0.05 were used to compare the differences between means. The significance of the correlation coefficient was analyzed using Pearson correlation.

## 5. Conclusions

In conclusion, this study has shown that the effect of Si on the quality of fragrant rice, as measured by the grain 2AP concentration, can differ markedly among varieties, while there were only slight differences in the yield response to Si among the fragrant rice varieties and the non-fragrant variety studied. KDML105 represents the varieties a with high 2AP concentrations when Si supply is low, with 2AP increasing only slightly with Si application. Varieties with low 2AP concentrations when the Si supply is limited may respond strongly to the 2AP concentration as shown with PTT1 or weakly as was the case of BNM4, which responds strongly to a moderate increase in Si supply in its 2AP concentration. The variation also includes the response to Si fertilizer in terms of gene expression associated with 2AP biosynthesis in the polyamine pathways, 2AP content, and the response of the *OsLSi6* gene to Si. The enhancement of 2AP production in fragrant rice under Si fertilizer application mainly occurred through the upregulation of *Badh2*, *DAO*, *ProDH*, and *P5CS* gene expression. An increasing proline concentration was not associated with an increase in 2AP concentration. Thus, for future work, confirmation of the varietal differences in the effects of Si on the aromatic compound 2AP would be useful for the management and breeding of fragrant rice. Elucidating the metabolites—precursors, products, and enzyme activities—in the 2AP biosynthesis pathways and not only the polyamine pathway but also the glycolysis pathway, along with the associated genes responsible for 2AP accumulation in the glycolysis pathway, coupled with investigations into mechanisms linked to silicon should be studied. Furthermore, these results could be valuable for rice breeders and farmers in upcoming studies exploring the association between phenotypic and genotypic traits or in developing a bi-parental population or multi-parental population. By doing so, researchers could gain a better understanding of the complex relationships within this pathway. This would not only provide breeders with unique and customized germplasm but also enable farmers to utilize high-yield lines. Furthermore, these studies could lead to the development of a set of interesting and unique markers for Thailand’s landrace populations. Additionally, they may contribute to the development of novel aromatic varieties responsive to silicon application, thereby enhancing grain aroma.

## Figures and Tables

**Table 1:** Yield component of four rice varieties grown at different silicon fertilizer applications. The samples were evaluated at maturity. The data are the means of three replications. (n = 24).

Variety (V)	Silicon Fertilizer (Si)	Plant Height (cm)	Number of Tiller Plant^−1^	Number of Panicle	Number of Spikelet Panicle^−1^	Filled Grains (%)	1000-Grain Weight (g)
BNM4	Si0	97.7 ± 3.13 ^d–f^	4 ± 0.31	4 ± 0.22	117.0 ± 6.11 ^f^	66.5 ± 3.89 ^h^	35.0 ± 0.74 ^bc^
	Si75	104.2 ± 2.20 ^bc^	4 ± 0.17	4 ± 0.26	142.3 ± 2.84 ^bc^	65.5 ± 1.46 ^h^	35.6 ± 1.50 ^b^
	Si150	99.3 ± 2.84 ^c–e^	4 ± 0.21	4 ± 0.17	150.2 ± 4.94 ^b^	75.5 ± 0.72 ^fg^	35.8 ± 0.75 ^b^
	Si225	98.7 ± 3.02 ^c–e^	5 ± 0.22	4 ± 0.26	142.7 ± 3.68 b^c^	78.9 ± 1.75 ^d–f^	35.0 ± 0.98 ^bc^
KDML105	Si0	110.0 ± 1.71 ^b^	4 ± 0.17	4 ± 0.17	133.8 ± 10.14 ^c–e^	78.4 ± 2.13 ^ef^	31.5 ± 0.38 ^fg^
	Si75	122.7 ± 0.92 ^a^	4 ± 0.21	4 ± 0.31	171.0 ± 4.90 ^a^	84.4 ± 1.77 ^b–d^	32.1 ± 0.42 ^e–g^
	Si150	125.8 ± 1.23 ^a^	4 ± 0.21	4 ± 0.31	169.3 ± 2.94 ^a^	86.8 ± 1.75 ^ab^	31.4 ± 0.25 ^fg^
	Si225	126.5 ± 1.98 ^a^	4 ± 0.21	4 ± 0.22	168.2 ± 4.28 ^a^	90.7 ± 1.11 ^a^	33.0 ± 0.81 ^c–f^
PTT1	Si0	97.7 ± 2.39 ^d–f^	4 ± 0.17	4 ± 0.17	101.7 ± 3.23 ^g^	80.0 ± 1.50 ^d–f^	32.3 ± 0.53 ^d–g^
	Si75	77.6 ± 1.45 ^g^	4 ± 0.21	4 ± 0.17	121.0 ± 4.20 ^ef^	85.9 ± 1.56 ^a–c^	33.3 ± 0.69 ^c–f^
	Si150	75.3 ± 1.25 ^g^	5 ± 0.26	5 ± 0.33	125.0 ± 2.12 ^d–f^	80.7 ± 1.23 ^c–f^	33.3 ± 0.85 ^c–f^
	Si225	74.4 ± 1.46 ^g^	5 ± 0.21	5 ± 0.22	128.8 ± 6.03 ^c–f^	79.1 ± 3.03 ^d–f^	34.2 ± 0.62 ^b–d^
SPR1	Si0	92.7 ± 2.02 ^f^	5 ± 0.22	4 ± 0.21	121.2 ± 6.81 ^ef^	66.6 ± 1.63 ^h^	29.0 ± 0.59 ^h^
	Si75	101.1 ± 2.24 ^cd^	4 ± 0.17	4 ± 0.17	141.8 ± 5.98 ^bc^	70.5 ± 1.32 ^gh^	30.4 ± 0.66 ^gh^
	Si150	94.2 ± 2.02 ^ef^	4 ± 0.21	4 ± 0.17	134.0 ± 4.49 ^c–e^	83.7 ± 1.33 ^b–e^	32.8 ± 0.65 ^d–f^
	Si225	98.4 ± 1.48 ^c–f^	4 ± 0.21	4 ± 0.17	136.2 ± 6.42 ^b–d^	82.2 ± 2.94 ^b–e^	33.8 ± 0.70 ^b–e^
Analysis of variance							
Variety (Var)		***	ns	ns	***	***	***
Silicon fertilizer application (Si)		ns	ns	ns	***	***	***
Var × Si		***	ns	ns	***	***	ns
LSD_0.05_ (V)		2.94	-	-	7.48	2.79	1.05
LSD_0.05_ (Si)		-	-	-	7.49	2.79	1.05
LSD_0.05_ (V × Si)		5.88	-	-	14.95	5.57	-

*** indicate significant difference at *p* < 0.05, *p* < 0.01, and *p* < 0.001, respectively, and ns indicates no significant difference. Different superscript lowercase letters indicate least significant differences within each column (*p* < 0.05).

**Table 2:** Correlation analysis of proline, 2AP concentration, and the relative gene expression levels (RQ) of *Badh2*, *DAO*, *OAT*, *ProDH*, and *P5CS* genes in 5DAF of four rice varieties grown at different silicon fertilizer applications.

	Proline Concentration (µmoles/g FW)	RQ*Badh2* Gene	RQ*DAO* Gene	RQ*OAT* Gene	RQ*P5CS* Gene	Rq*prodh* Gene
RQ*Badh2* gene	0.17 ^ns^					
RQ*DAO* gene	0.20 ^ns^	0.34 *				
RQ*OAT* gene	0.41 *	0.11 ^ns^	0.59 ***			
RQ*P5CS* gene	0.44 *	0.44 **	0.45 ***	0.26 ^ns^		
RQ*ProDH* gene	0.34 *	0.22 ^ns^	0.06 ^ns^	−0.05 ^ns^	0.23 ^ns^	
2AP concentration (ppm)	0.16 ^ns^	0.30 *	0.34 *	−0.00 ^ns^	0.68 ***	0.38 **

RQ = Relative quantification in gene expression; *, **, and *** indicate significant difference at *p* < 0.05, *p* < 0.01, and *p* < 0.001, respectively, and ns indicate no significant difference.

**Table 3:** Primer list used for studying gene expression levels.

Genes	Primer		References
*OsLsi6*	F:	AGATCGTCGTCACCTTCAACAT	[25]
	R:	CTTGAAGGAGGAGAGCTTCTGG	
*DAO*	F:	TGGCAAGATAGAAGCAGAAGT	[44]
	R:	GTCCATACGGGCAACAAA	
*Badh2*	F:	TGTGCTAAACATAGTGACTGGA	[51]
	R:	CTTAACCATAGGAGCAGCT	
*OAT*	F:	ATGAAATGATGTTGCCGATGA	[84]
	R:	CCTAATGTCCGACCATGAAAA	
*ProDH*	F:	ATTGCTCTCGTCTTCCTCCT	[85]
	R:	ATGACTCGATCGCTTCACTC	
*P5CS*	F:	TGGCAATTCGAAGTGGTAAT	
	R:	AGCAAATCTGCGATCTCATC	
*OsActin* *(housekeeping gene)*	F:	GACTCTGGTGATGGTGTCAGC	[51]
	R:	GGCTGGAAGAGGACCTCAGG	

## Data Availability

Data are contained within the article.
